# A Rare Case of Idiopathic Bilateral Medial Canal Fibrosis

**DOI:** 10.7759/cureus.41830

**Published:** 2023-07-13

**Authors:** Farhana Kamaruzaman, Mohd Irman Shah Ibrahim, Asma Abdullah

**Affiliations:** 1 Otorhinolaryngology - Head and Neck Surgery, Universiti Kebangsaan Malaysia Medical Centre, Kuala Lumpur, MYS; 2 Otolaryngology - Head and Neck Surgery, Hospital Sultanah Nur Zahirah, Kuala Terengganu, MYS; 3 Otolaryngology - Head and Neck Surgery, Universiti Kebangsaan Malaysia Medical Centre, Kuala Lumpur, MYS

**Keywords:** pseudomembrane, idiopathic, fibrous tissue, external ear, medial canal fibrosis

## Abstract

Medial canal fibrosis usually occurs as sequelae of known conditions such as trauma, infection, or surgery. Rarely, it occurs without an identifiable cause, hence the term idiopathic medial canal fibrosis. Regardless of the etiology, the reportedly most successful treatment is surgery. A 52-year-old lady presented to us with bilateral reduced hearing and left ear tinnitus. There was no significant history to suggest the possible cause of the symptoms. Clinically, there is the presence of thick solid fibrous in the bilateral ear canal. Audiological examination revealed a conductive hearing loss bilaterally with tympanometry of type B, and imaging was done. She successfully underwent canalplasty following that and is doing well to date. In this paper, we reported a rare case of bilateral idiopathic medical canal fibrosis, and we discuss the causes, diagnosis, and best treatment for this disease.

## Introduction

Medial canal fibrosis is the formation of thick, solid fibrous tissue in the medial part of the bony external ear canal, which can be partial or complete [1]. Most patients presented with hearing loss (78%) and otorrhea (59%) [2]. The most common causes are recurrent infections or inflammation (56%), followed by iatrogenic (37%) and idiopathic (7%) causes [2]. There are two stages of medial canal fibrosis, the wet and the dry. In the wet phase, the symptoms of otorrhea and ear fullness will be present due to inflammation of the ear canal, which subsequently heals with progressive fibrosis and stenosis. In the dry phase, the main symptoms are conductive hearing loss and dry ears. The management of this disease can be medical or surgical treatment. Medical therapy is a choice for patients in the wet phase while surgery is for patients in the dry phase [3].

## Case presentation

**Figure 1:**
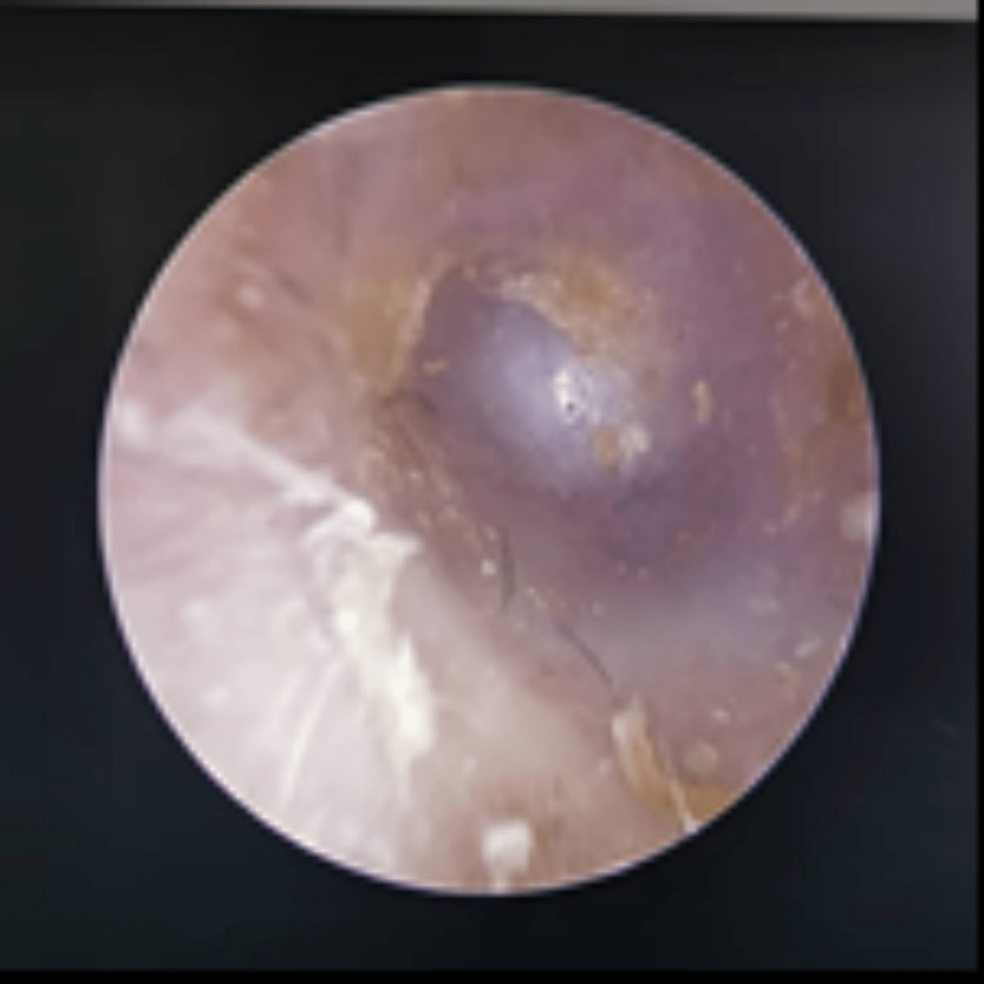
Left otoscopy examination showing a shortened left external ear with a pseudomembrane

**Figure 2:**
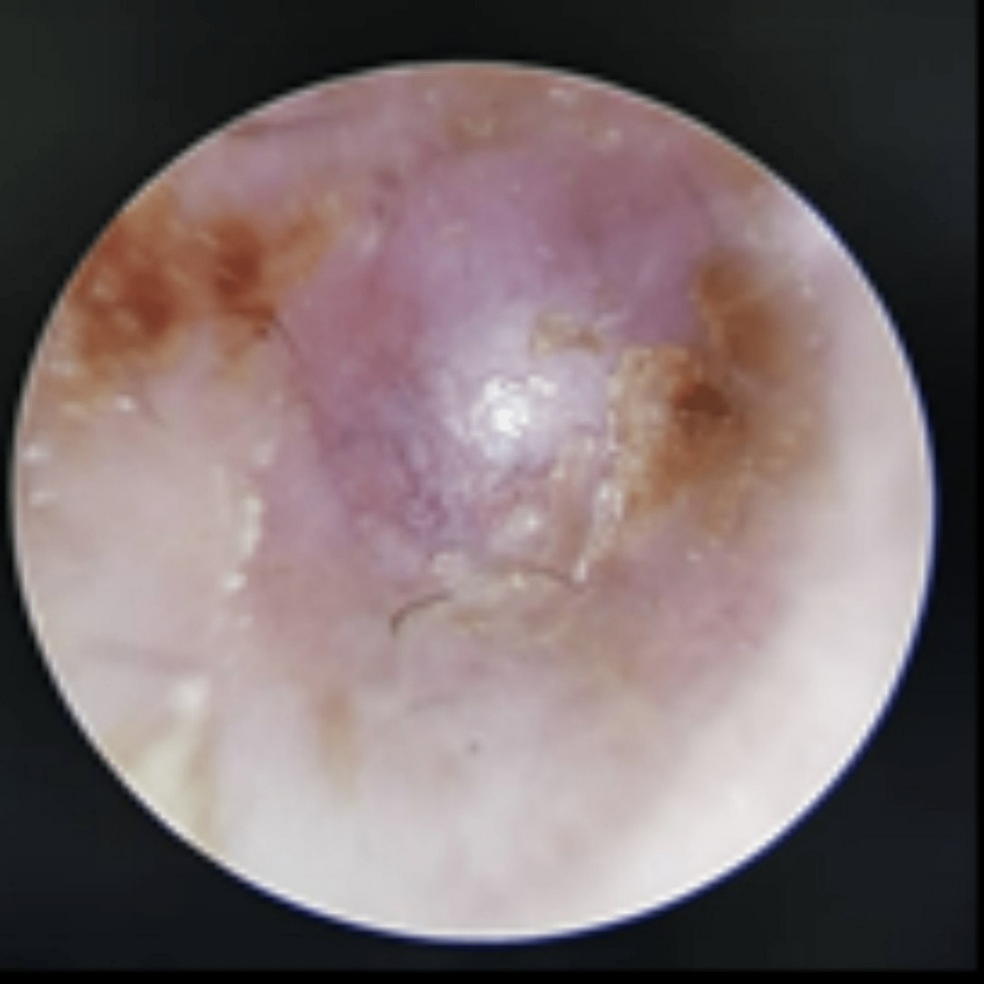
Right otoscopy examination showing a shortened right external ear with a pseudomembrane

**Figure 3:**
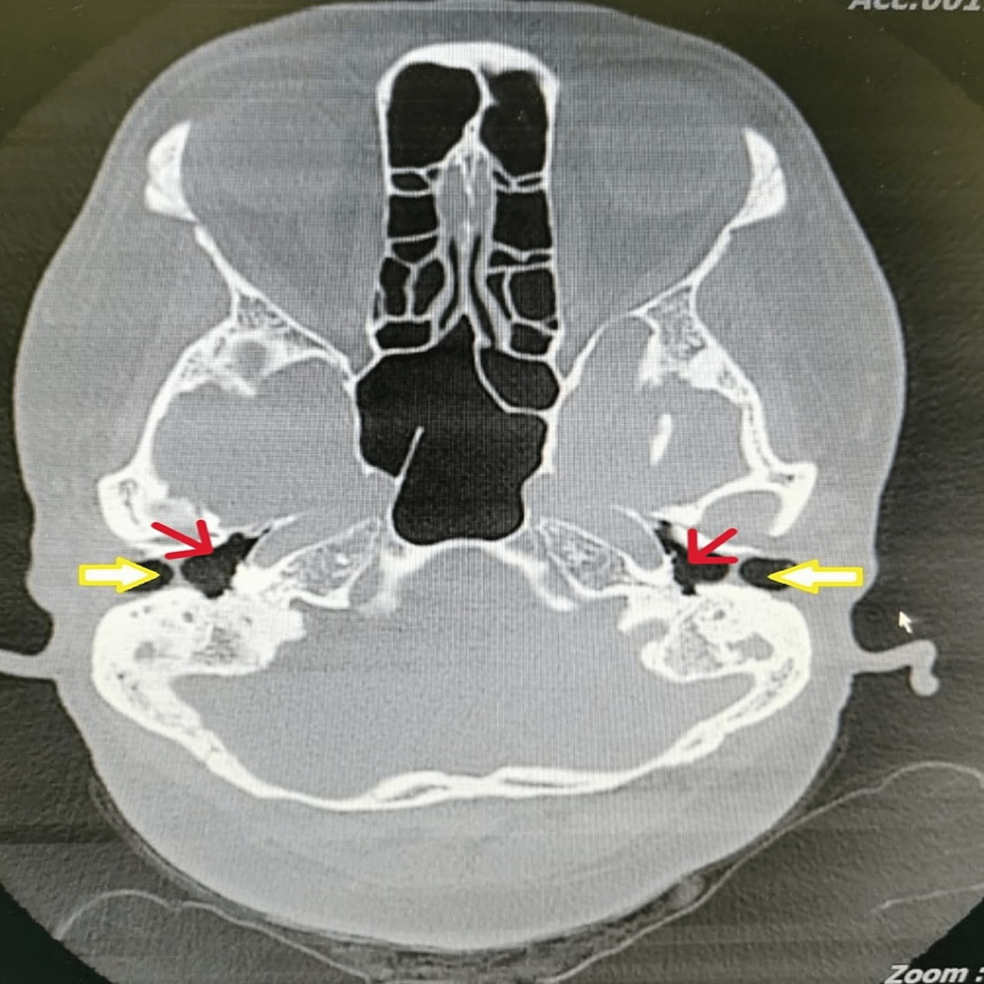
HRCT temporal bone shows crescentic soft tissue thickening (yellow arrow) overlying lateral to the tympanic membrane (red arrow) HRCT: high-resolution computed tomography

A 52-year-old Malay lady, previously well with no known medical illness, was referred to our clinic with a complaint of left ear tinnitus and bilateral decreased hearing for one year. She denied any other symptoms such as otalgia, otorrhea, chronic itchiness of ears, ear digging, history of trauma, or any ear surgery and nasal symptoms. Otological examination revealed a shortened bilateral ear canal with the presence of a thick solid fibrous in the medial part of the bone external auditory canal (Figures 1, 2). A pure tone audiometry test showed bilateral moderate to severe mixed hearing loss. A high-resolution computed tomography (HRCT) temporal bone was performed (Figure 3) and the diagnosis of bilateral complete medial canal fibrosis was made based on history, clinical, and audiometry. She underwent left canalplasty with a temporalis fascia graft. Intraoperatively, there was the presence of a thick solid fibrous blend with the left tympanic membrane. The tympanic membrane perforated 25% and the middle ear looked healthy. The rupture of the tympanic membrane was repaired. She followed up well to one-month post-surgery. She is planning to undergo another surgery on the right ear later.

## Discussion

Medial canal fibrosis (MCF) is a rare otological condition [4]. The findings and their clinical correlations are still hypothetical in view of the rarity of this condition and the absence of large studies and clinical trials on this disease [3].

MCF has been classified based on its etiology, for instance, post-inflammatory, traumatic, or postoperative complications [4]. It is usually caused by recurrent infections or inflammation (56%), followed by iatrogenic (37%) and idiopathic causes (7%) [2]. Infection or inflammation leads to the formation of fibrotic scar tissue and the extension of this fibrotic tissue toward the cartilaginous junction of the external auditory canal (EAC) [2,5]. The idiopathic cause is possibly due to the loss of the squamous epithelium on the lateral surface of the tympanic membrane and leads to the exposure of its fibrous layer [6]. Ongoing inflammation and healing occur through granulation tissue formation. This process will make stenosis of the ear canal continue up to the area of the bony cartilaginous junction [6]. In this case, the differential diagnosis includes external auditory canal cholesteatoma, gummatous lesions of tertiary syphilis, lupus erythematosus, and primary carcinoma of the external auditory meatus. Histology should be performed in all cases; however, no histology sample was taken in this case. Based on history, this patient has no preceding infection, trauma, or any surgical intervention recorded. It was supported by clinical examination upon presentation, which revealed no signs to suggest recent infection or trauma in the EAC.

MCF is characterized by two stages of the disease. The first is the wet phase. In this phase, the inflammation process is ongoing and subsequently develops fibrosis and stenosis of the EAC. The patient will present with recurrent otorrhea and aural fullness. Later, a non-discharging stenotic ear canal with dense fibrosis presents. Otologic findings can show shortened EAC with a pseudomembrane. Pure tone audiometry (PTA) usually shows conductive hearing loss in view of the sound being unable to reach the tympanic membrane [1]. In our case, the patient presented with a dry phase, and the findings of the otoscopy were suggestive of MCF. PTA showed bilateral moderate to severe mixed hearing loss. The aging factor is also one of the causes of mixed hearing loss.

High-resolution computed tomography (HRCT) of the temporal bone in MCF showed a normal middle ear with the presence of a crescentic area of soft tissue thickening overlying the lateral tympanic membrane surface and extending laterally to fill the medial EAC [7]. However, there were no bony erosive changes [7]. HRCT of temporal bone findings in this patient showed fibrotic tissue at the lateral part of the tympanic membrane; however, it seems there is a space between the tympanic membrane and the fibrotic area.

The best treatment for the dry phase of MCF is surgical [2]. The main goals of the surgery are to re-establish a patent epithelium-lined ear canal for good sound conduction, and self-cleaning function [8]. The surgical procedure involves the removal of the fibrotic tissue, canaloplasty, and skin grafting [8]. The previous study had done and compared their results regarding recurrence rate and hearing improvement respectively, with their years of follow-up with other literature reviews [2]. Their studies reported more than 50% of cases had improved hearing [2]. MCF cases treated with simple excision of fibrous tissue without split skin graft (SSG) have a 100% failure rate. Compared with excision with SSG, 70% have a patent meatus with 79% hearing improvement [9]. In the present case, a canalplasty with temporalis fascia graft was performed on the left ear. There is no previous study reported regarding using the temporalis fascia as a graft. However, this case was suggested by our consultant otologist based on their experiences who found that the healing process by using temporalis fascia is faster and lined the canal well. She was well during the postoperative follow-up in the clinic one-month post-surgery and is planning to undergo surgery on the right ear too.

## Conclusions

Idiopathic medial canal fibrosis is a rare otological condition with unfathomable etiology. It can affect a patient's hearing and life. The diagnosis should be explored, and idiopathic medial canal fibrosis can be successfully treated by surgery as proven by our case.
